# Cost Evidence Yields the Viability of Metal Oxides Synthesis Routes

**DOI:** 10.1021/acssuschemeng.5c06752

**Published:** 2025-10-07

**Authors:** Despina A. Gkika, George Z. Kyzas

**Affiliations:** Hephaestus Laboratory, School of Chemistry, Faculty of Sciences, 37791 Democritus University of Thrace, GR 65404 Kavala, Greece

**Keywords:** metals, nanomaterials, economic analysis, sensitivity analysis, atom economy

## Abstract

Metal-based nanomaterials continue to be extensively studied, as they are regarded as the foundation of significant technological advancements due to their promising properties. However, despite these advantages, their broad adoption remains constrained by the high costs associated with the synthetic methods commonly reported in the literature. The novelty of this study lies in its integrated approach, which combines activity-based costing, total cost of ownership, and green metrics (including percentage yield, stoichiometric factor, atom economy, and reaction mass efficiency). Using three illustrative case studies (TiO_2_, Al_2_O_3_, CeO_2_) it was shown that if the synthesis processes were considered cost alone, the TiO_2_ resulted in the lowest total synthesis cost. Green metrics evaluation further reinforce the sustainability of TiO_2_. A comparative assessment of green metrics for TiO_2_ and Al_2_O_3_ revealed that while TiO_2_ and Al_2_O_3_ exhibit comparable atom economies (TiO_2_: 19.37%; Al_2_O_3_: 19.40%), TiO_2_ achieves a higher percentage yield (97 vs 95%) and significantly outperforms Al_2_O_3_ in terms of stoichiometric factor (8.51 vs 25.77), indicating more efficient use of reactants and reduced chemical waste. Additionally, TiO_2_ shows a marginally higher Kernel’s Reaction Mass Efficiency (18.79 vs 18.43%). The findings indicate that low cost and efficiency are closely interconnected concerns in synthetic routes.

## Graphical abstract

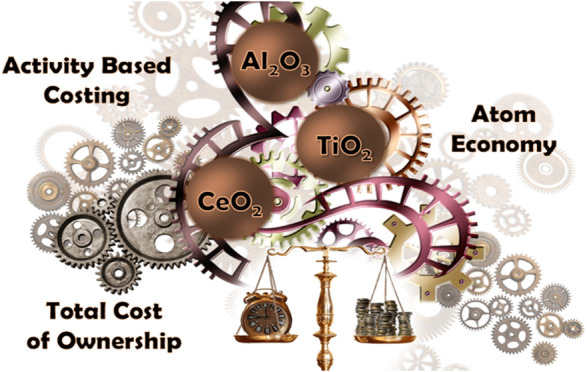

## Introduction

The synthesis of nanomaterials is a field that has seen remarkable growth in recent years. The synthesis process is considered a critical strategy for the successful discovery and development of new materials.^1^ In particular, nanometals have been the focus of extensive research.^2^ Among various metals, titanium dioxide,^3^ mesoporous alumina^4^ and cerium oxide^5^ have attracted significant attention. For example, TiO_2_ is valued for its strong oxidation potential in pollutant decomposition, its physical and chemical stability, and its relatively low cost and toxicity.^6^ Similarly, mesoporous silica and mesoporous alumina have gained considerable interest due to their high specific surface area, large pore volume, and excellent stability.^4^ Additionally, cerium oxide (CeO_2_), an important rare earth metal oxide, has received increasing attention in recent decades because of its wide-ranging applications in catalysis, pollution reduction, and other fields. This is mainly due to its distinctive redox behavior, which involves the storage and release of oxygen under both oxygen-rich and oxygen-deficient conditions.^4^

The synthesis of metal oxide nanoparticles with controlled shape and size is of both fundamental and technological importance. These particles play a crucial role in understanding nanoscale physical phenomena and are applied in various areas such as optics, catalysis, energy, and microelectronics.^7^ The desirable properties of metal nanomaterials continue to drive efforts toward developing cost-effective synthetic methods. A variety of methods, both bottom-up and top-down, are used to create metal and metal oxide nanomaterials. These techniques, such as wet chemical methods, hydrothermal synthesis, templating, thermal decomposition, pulsed laser ablation, microwave-assisted synthesis, chemical vapor deposition, combustion, gas-phase techniques, sol–gel processes, and solvothermal synthesis, each have their own unique benefits and drawbacks.^8^ Thus, the green and sustainable production of chemicals is a critical element in the transition from a linear economy, which consumes natural resources and degrades ecosystems, to a circular economy that is resource-efficient and designed to eliminate waste. To enable this transformation, reliable metrics are required to compare the greenness and sustainability of competing technologies.^9^ Green chemistry metrics provide quantitative tools for evaluating the environmental impact, efficiency, and sustainability of chemical processes and products.^10^

However, the discovery and optimization of synthesis protocols for nanomaterials demand a highly skilled and trained workforce.^11^ In this context, synthesis strategies and conditions, such as pretreatment, washing, and storage, can significantly influence the overall cost of the process. Developing low-cost synthesis protocols for nanomaterials remains a major bottleneck and is therefore an area of ongoing research and development.^12^ There is a clear need to develop low-cost synthesis methods that eliminate the complexity associated with managing synthesis expenses. Although the cost of synthesis is a significant concern, economic considerations are often an afterthought during the initial stages of chemical synthesis design.^13^ While several established methodologies exist,^14,15^ recent research has primarily focused on nanomaterial performance, with economic factors related to synthesis cost seldom discussed in the scientific literature. To evade these drawbacks, Gkika et al. proposed a cost model. Reflecting growing scientific interest, one study carried out an economic analysis to evaluate the effect of energy costs on the total synthesis cost of membranes made from polymers of intrinsic microporosity.^16^ In another study involving chitosan-based adsorbents, labor cost was identified as the most significant contributor to the overall synthesis cost.^17^ Although these studies set the precedent for reducing synthesis costs, the generalizability of their findings is unclear. Each synthesis route may present distinct economic vulnerabilities, which can ultimately increase the total cost. Experimentally validated synthetic pathways can be compared using quantitative metrics, such as yield and atom economy, or qualitative factors, such as strategic design and novelty.^18^ Considering these issues, and in light of the many confounding variables involved, it is important to thoroughly understand the individual cost factors that influence a synthetic process. Comparing different nanomaterial synthesis routes using quantitative metrics is nontrivial.

Our goal is to create a measure of route efficiency in terms of cost. Since this information is rarely available in a direct form, we use various complexity metrics related to metal materials as a surrogate. In our previous research, different synthetic routes of the same material (rGO) were explored through synthesis.^19^ Inspired by the intrinsic advantages of the synthesis cost profile in this contribution, we further explore a large economic scope of different metal nanomaterials and studied their cost relationships. The primary objective is to establish an integrated methodology that can elucidate the intricate economic and environmental profiles of different metal oxide materials, while also accounting for the complexities of their synthetic route characteristics. The novelty of this work lies in its integrated framework, which combines Activity-based costing (ABC) model,^20^ Total cost of ownership (TCO) model,^21^ and green metrics to provide detailed insights into the key cost and environmental-driving factors. This multidisciplinary approach is pivotal in synthetic processes, as it encompasses the technologies and techniques employed, the sustainable pathways to be considered, the associated economic costs, and the environmental impacts, while employing a metal materials complexity metric as a surrogate.

We began with an early stage economic assessment using the TCO model^21^ and activity based costing (ABC) model.^20^ These strategic cost management tools measure the cost and efficiency of activities, helping to reveal the dynamics behind three different synthesis routes. This comprehensive, experimentally supported cost analysis offers crucial insights into how various factors impact total synthesis costs, ultimately guiding the selection of a specific metal nanomaterial. By evaluating the cost implications of different synthetic outcomes, this analysis enables the identification of a robust process-cost framework, which is essential for implementing sustainable synthesis practices at the laboratory scale. Additionally, we used the green metrics as a macro-level sustainability strategy to assess efficiency aspects of the synthetic routes. This method helps uncover how cost is influenced by changes in the choice of metal nanomaterials, providing a clearer picture of the cost-efficiency landscape. Overall, the proposed methodology has the potential to significantly reduce synthesis costs while enhancing process efficiency.

This framework aims to provide a strategic management perspective at the laboratory scale. The economic dimension, in particular, is expected to act as a catalyst for a financially sustainable paradigm shift. Strengthening the ability to assess cost implications and efficiency will enhance research planning and enable informed perspectives on nanomaterials development, thereby supporting future innovation. By examining the relationship between cost and green metrics across diverse materials, this comprehensive, experimentally based cost analysis offers critical insights into how economic evidence yields viability and sustainability in a synthetic pathway.

## Experimental Procedures

The studied materials were selected according to previous work.^22^

### Synthesis of Cerium Oxide^23^

CeO_2_ nanoparticles were synthesized using a reverse micelle method. Phosphatidylcholine was dissolved in toluene to form micelles, into which cerium nitrate solution was added and stirred. Ammonium hydroxide was then titrated to initiate nanoparticle formation. After 45 min, CeO_2_ nanoparticles formed and were collected via centrifugation, followed by sequential rinsing with methanol, ethanol, and water. The nanoparticles were dispersed in sodium citrate solution, ultrasonicated until clear, pH-adjusted to 7.4, and sterilized by filtration. The final yield was about 50 mg of stabilized CeO_2_ nanoparticles in 100 mL of solution.

### Synthesis of Mesoporous Alumina^24^

Mesoporous aluminas (MAs) were synthesized by hydrolyzing aluminum isopropoxide (with or without added inorganic aluminum salts) in hot water, followed by acid addition and stirring at 85 °C for 12 h. During the first 2 h, isopropanol was allowed to evaporate. A structure-directing template was then added and the mixture was stirred at room temperature for 12 h. The product was dried at 70 °C and calcined at 700 °C. To study the impact of template type and concentration, four surfactants (P123, F127, CTAB, SDS) were tested, along with three aluminum salts (Al­(NO_3_)_3_, Al_2_(SO_4_)_3_, AlCl_3_) to assess precursor and anion effects. Samples were labeled as MAxAyAlB, denoting template type, amount, and aluminum precursor used. For instance, MA1P15AlN indicates use of 1 g P123 and 15 mol % Al­(NO_3_)_3_.

### Synthesis of Titanium Dioxide^25^

TiO_2_ nanoparticles were synthesized using titanium butoxide (Ti­(OBu)_4_) and anhydrous alcohol. A 1:1 mixture was ultrasonically dispersed, then water was added dropwise while stirring for 2 h at pH 3.0. The solution was aged for 24 h, filtered, and washed with deionized water and alcohol. After drying at 100 °C for 12 h, the resulting precursor was calcined at either 500 or 650 °C for 2 h in air to yield TiO_2_ nanoparticles.

#### Framework

This research aims to evaluate low-cost approaches at the lab scale. The synthesis process is divided into two main categories: (i) planning and (ii) design. It is further organized by tasks and methods, outlining step-by-step activities for achieving the research objectives. According to Gkika et al.,^16^ the complexities of managing the synthesis process can only be fully understood through activity-based costing (ABC) and other detailed assessments. Furthermore, the application of green chemistry principles (GCPs) can further enhance economic performance. GCPs can be grouped into three main areas: Input selection and reduction encompasses 1 (waste prevention), 2 (atom economy), 7 (use of renewable feedstocks), 8 (reduction of derivatives), and 9 (catalysis). Sustainable design involves 4 (designing safer chemicals), 6 (energy efficiency), and 10 (design for degradation).Safety management includes 3 (less hazardous chemical synthesis), 5 (safer solvents and auxiliaries), 11 (real-time pollution prevention analysis), and 12 (inherently safer chemistry for accident prevention).^26^ The application of green chemistry principles across the two synthetic pathways is outlined in Table 1.

**Table 1:** Application of Green Chemistry Principles and ABC Costing in Two Synthetic Pathways

planning	synthesis process strategy	objectives	green chemistry principle	category	economic performance
	synthesis of titanium dioxide	1. green chemistry	#2	atom economy	ABC method, TCO
	synthesis of mesoporous alumina	2. economic performance of the synthetic routes	#2	atom economy	ABC method, TCO
designing	setting the problem into interconnecting tasks				
	develop the tasks according to the objectives				

#### Activity-Based Total Cost of Ownership (ABC-TCO) Process

According to Gkika et al.,^16^ the complexities of synthesis management can only be fully captured through activity-based costing (ABC) and similar detailed evaluations. Models such as activity-based life cycle costing (ABC-LCC) and activity-based total cost of ownership (ABC-TCO) (Figure 1) provide a more accurate and comprehensive framework for assessing the full range of costs associated with investment decisions and purchasing activities.^27^

**Figure 1:**
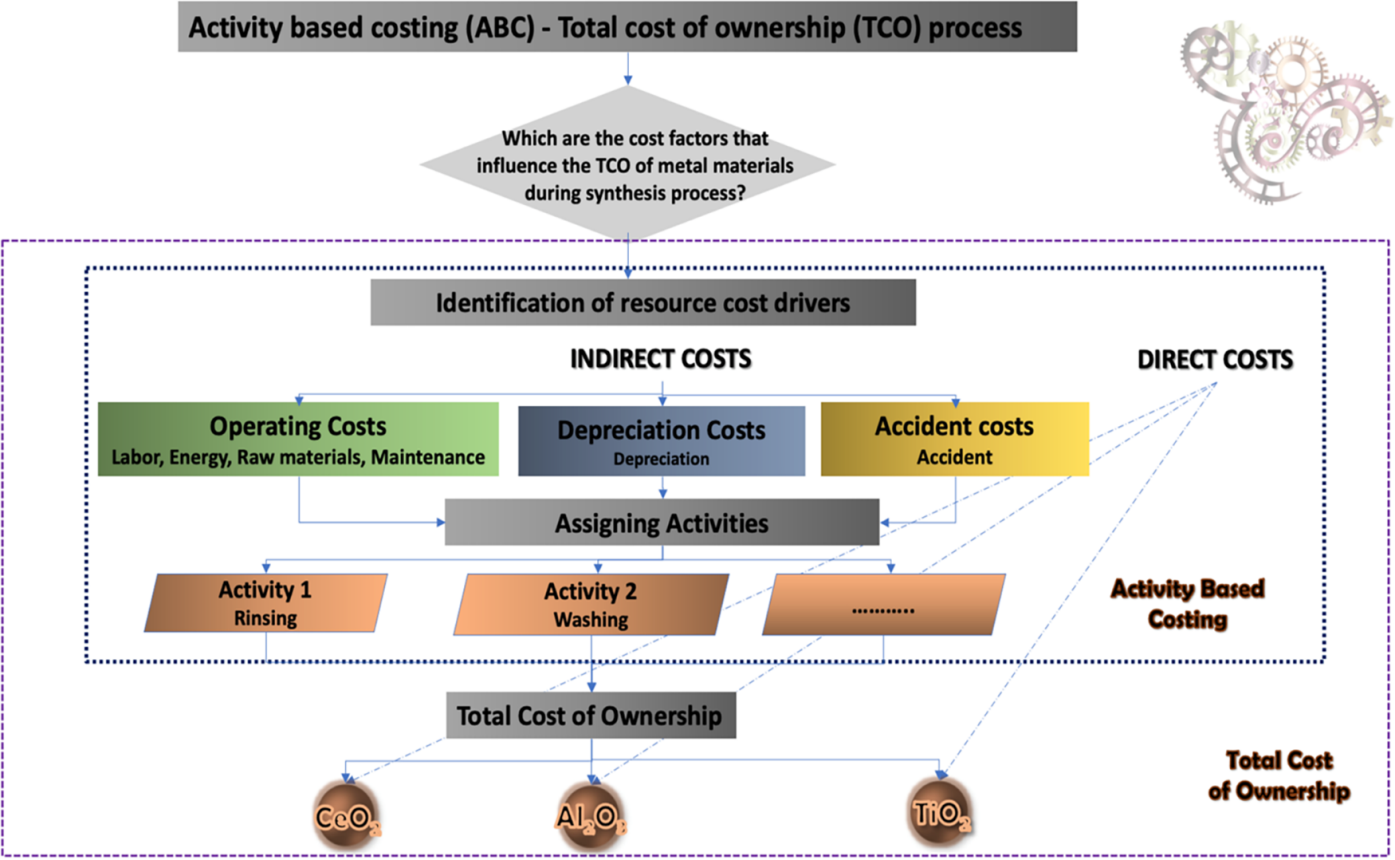
ABC-TCO process.

#### Activity Based Costing (ABC) Method

From a theoretical standpoint, ABC analysis, as described by Cooper and Kaplan,^28^ offers a viable alternative to traditional cost accounting methods. Following Lewis,^29^ ABC is defined as a technique that accumulates product costs by identifying all activities necessary for manufacturing the product. Consequently, the total cost of a product equals the sum of raw material costs and the total costs of all production-related activities. ABC primarily focuses on the root causes of indirect costs. Unlike traditional costing methods, ABC defines cost categories as activities rather than production cost centers.^30^ Additionally, the cost drivers used to assign activity costs differ structurally from those in conventional costing systems.^29^ According to Cooper and Kaplan^28^ ABC can generally be described as a two-step process. In the first step, activities, such as processing material orders, marketing, or handling customer orders, are identified. They require resources including personnel, equipment, materials, and capital. The costs associated with each activity’s resource use are calculated based on resource cost drivers. In the second step, ABC assigns these activity costs to the relevant cost objects, such as products or services. To determine the cost structure of an object, various activities are linked to it through activity cost drivers. Thus, activity cost drivers and resource cost drivers serve as the crucial connection between the cost object, the associated activities, and the resources utilized.^30^

#### Supported Nanoparticle Cost Estimation and Input Parameter Choices for TCO Model

This section outlines the calculation of each cost component and the key input parameters used in modeling the synthesis process. The analysis goes beyond chemical costs to include materials, labor, maintenance and energy. Estimates were generated using Excel (v16), with all assumptions and variables detailed in Supporting Information. Prices are reported in Euros. A widely accepted definition of the TCO concept from^21^ provides a starting point: TCO is an activity-based approach that utilizes principles of ABC to identify and analyze costs. This definition highlights two main aspects: (a) TCO is rooted in activity-based methodology, and (b) it functions as a tool for cost analysis. This conceptual framework underpins our research and is grounded in both academic rigor and widespread recognition within the field. The total cost is the sum of costs for materials, labor, accidents, energy, maintenance, and depreciation. Raw material prices were estimated by combining vendor quotations, publicly available and proprietary price databases, and input from industry experts. Multiple sources were consulted to verify and average each assumed price. To calculate precursor usage per gram of product, both the amount of precursor used per synthesis and the synthesis yield are needed. Precursor quantities were taken from published protocols, while yield data, in grams of product, came from reported sources. All synthesis protocols were taken from referenced literature. Labor costs for synthesis were estimated either as the total hours required to complete the process or as the actual active labor time. It is important to distinguish between these two, since total hours include activities like literature review, waiting times, and personnel off-duty periods. Adding these extra hours inflates labor costs, so this study considers actual active hours as the more accurate measure. Maintenance and accident costs are detailed in Supporting Information. For accident costs, replacement expenses were included in the calculations. Synthesis costs were estimated using both ABC and TCO methodologies. The model is structured into three tiers: inputs, calculations, and outcomes, with results presented in numerical or graphical format

1 $$\mathrm{TCO}=C_{R}+C_{E}+C_{L}+C_{M}+C_{D}$$

where: TCO is the total cost of ownership of the synthesis process, *C*_R_ is the raw material cost, *C*_E_ is the energy cost, *C*_L_ is the labor cost, *C*_M_ is the maintenance cost, *C*_D_ is the depreciation of apparatus and equipment. The TCO model has been described previously.^19^

#### Sensitivity Analysis

To evaluate the relative importance of different input parameters on the synthesis cost for all materials, a sensitivity analysis was conducted. The results of this analysis were illustrated using tornado charts that depict the baseline scenario alongside variations in key parameters. The parameters examined in this study include: (i) raw material cost (base case ± 20%), (ii) accident cost (base case ± 20%), (iii) energy cost (base case ± 20%), and (iv) labor cost (base case ± 20%). Sensitivity data detailed in Supporting Information.

#### Atom Economy

Although selectivity and yield are key priorities in fine chemical research and academic settings, the efficient use of reactants – viewed through the lens of atom economy, is frequently neglected.^31^ Atom economy,^32^ is a key principle of green chemistry and one of the most widely used metrics for evaluating the efficiency of a process or synthesis. This concept essentially measures the proportion of reactant atoms that are incorporated into the final desired product(s). Atom economy is not merely a theoretical idea but serves as a practical guideline that helps scientists design and execute more efficient syntheses. The concept was formalized by Sheldon,^32^ who defined atom economy as the percentage of atoms utilized, calculated by dividing the molecular weight of the desired product by the total molecular weight of all products generated in the reaction.^33^

2 $$\mathrm{AE}\left(\%\right)=\frac{\mathrm{molecular}\;\mathrm{weight}\;\mathrm{of}\;\mathrm{product}}{\sum \mathrm{molecular}\;\mathrm{weight}\;\mathrm{of}\;\mathrm{reactants}}\times 100\%$$

Atom economy has been used to evaluate efficiency aspects of synthetic routes. This concept is highly logical and can be automated, provided that fully atom-mapped synthetic sequences are available, including reagents, although these mappings may sometimes lack accuracy. However, such detailed atom economy analyses are not routinely applied or reported when evaluating synthetic routes.^34^ Atom economy data detailed in Supporting Information section.

#### Percentage Yield

Percentage yield (Y) has long been regarded as a standard measure of reaction efficiency. Yet, it offers only a partial view of process performance. This limitation arises because (i) it treats the reactants as if they were isolated systems, which is rarely the case in practice, and (ii) it neglects the contribution of excess reagents. To obtain a more accurate picture of process greenness, additional efficiency metrics must therefore be considered.^35,36^

3 $$Y\left(\%\right)=\frac{\mathrm{actual}\;\mathrm{yield}}{\mathrm{theoretical}\;\mathrm{yield}}\times 100\%$$

Since atom economy expresses the proportion of reactant atoms incorporated into the desired product, that is, the efficiency of atom utilization within a chemical reaction, a higher percentage yield is typically linked to a more atom-efficient process, thus establishing a direct relationship between the two indicators. Comparing atom economy with the stoichiometric factor further highlights how reaction stoichiometry influences the effective use of atoms.^35^

#### Stoichiometric Factor

Stoichiometry describes the theoretical efficiency of atom use, while the stoichiometric factor (SF) provides a measure of the additional reagents consumed in practice.^35^ A SF value of 1 indicates that the reactants are present in exact stoichiometric proportions, with no excess reagents required. Values higher than 1 indicate that additional reagents are used in excess of the theoretical requirement, which reduces efficiency and generates more waste.^35,37,38^

4 $$\mathrm{SF}=1+\frac{\mathrm{total}\;\mathrm{mass}\;\mathrm{of}\;\mathrm{excess}\;\mathrm{reagents}}{\mathrm{total}\;\mathrm{stoichiometric}\;\mathrm{mass}\;\mathrm{of}\;\mathrm{reagents}}\times 100\%$$

5 $$\mathrm{SF}=1+\frac{\mathrm{atom}\;\mathrm{economy}\times \mathrm{total}\;\mathrm{mass}\;\mathrm{of}\;\mathrm{excess}\;\mathrm{reagents}}{\mathrm{theoretical}\;\mathrm{mass}\;\mathrm{of}\;\mathrm{the}\;\mathrm{product}}\times 100\%$$

#### Reaction Mass Efficiency (RME)

Reaction mass efficiency (RME) is often regarded as a more refined version of atom economy, since it integrates both reaction yield and the penalty imposed by excess reagent consumption.^39^ In this sense, RME provides an experimental assessment of how effectively reactants are incorporated into the final product.^40^ A higher RME value signifies improved process efficiency. Notably, the literature emphasizes RME as the most robust of the green metrics for quantifying and mitigating waste generation.^35^ In this work, RME was calculated according to two methodologies: Kernel’s RME (maximum RME) and Curzon’s RME^38,41–43^ .

6 KernelRME(%)=atomeconomy×yield

7 $$\mathrm{Curzon’s}\;\mathrm{RME}\;\left(\%\right)=\mathrm{atom}\;\mathrm{economy}\times \mathrm{yield}\times \frac{1}{\mathrm{SF}}\,$$

8 $$\mathrm{RME}=\frac{\mathrm{molar}\;\mathrm{mass}\;\mathrm{of}\;\mathrm{product}}{\sum_{i}\mathrm{molar}\;\mathrm{mass}\;\mathrm{of}\;\mathrm{reactant}\;i}\times \%\;\mathrm{yield}$$

Stoichiometric factor and reaction mass efficiency data are detailed in Supporting Information.

## Results and Discussion

### Benchmarking Economic Analysis of Synthetic Routes

We conducted an early stage economic assessment of the synthesis processes for three different nanometals: titanium dioxide, mesoporous alumina, and cerium oxide. This analysis employed ABC and TCO to evaluate the influence of various cost drivers on the overall synthesis process. All cost estimates are presented in Euros, using 2025 as the reference year for pricing. The analysis began by calculating the total synthesis cost for each nanomaterial. Breaking this total into individual cost components allowed for the identification of areas where cost reductions may be possible.

The analysis demonstrates that the TCO/ABC model reveals the main factors affecting costs during the synthesis process. Figure 1 illustrates the cost contributions of each component to the overall TCO for the three metal oxide nanoparticle synthesis pathways. Figure 2 presents the five largest cost components associated with each synthesis method.

**Figure 2:**
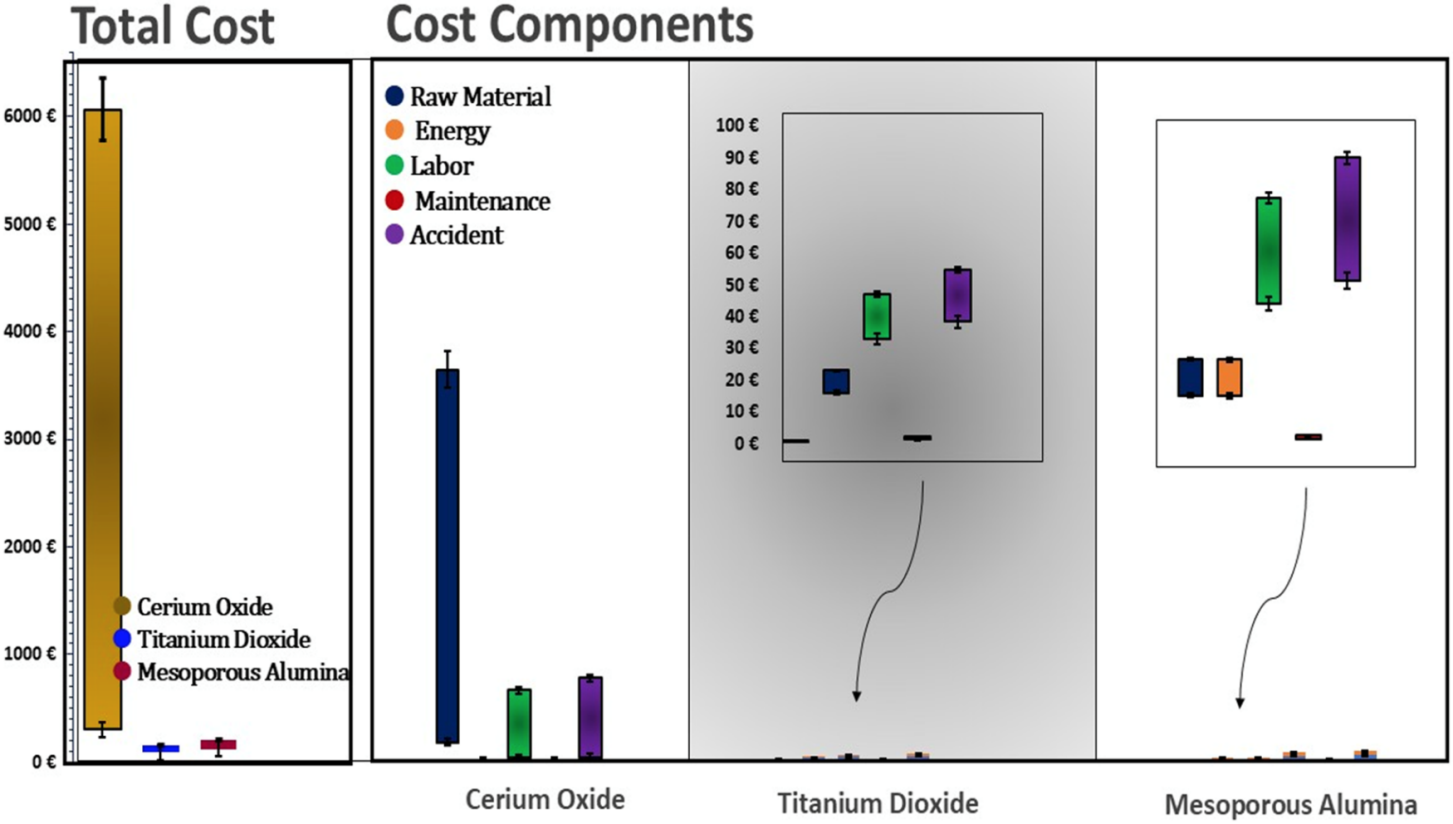
Distribution of TCO cost factors per studied synthesis process.

A key insight from this combined experimental and economic approach is that the material cost for cerium oxide is notably higher than that of titanium dioxide and mesoporous alumina. A key observation from Figure 2 is that the combined experimental and economic approach shows raw material cost as the dominant factor in the CeO_2_ TCO, while labor and accident-related costs are the primary contributors to the higher TCO in the TiO_2_ and Al_2_O_3_ synthesis process. In particular, for cerium oxide, more than 90% of the total synthesis cost is attributed solely to the cost of raw materials. The proportions of depreciation (ranging from 0 to 3.16%), maintenance (0 to 2.95%), and energy costs (0 to 0.78%) are relatively consistent across all nanomaterials.

Table 2 presents an overview of the TCO values for the materials under study, showing titanium dioxide with the lowest and cerium oxide with the highest TCO. Table 2 further indicates that energy costs can play an important role for all three materials, with a varying impact on their TCO. In contrast, maintenance costs remain low, resulting in only a small share of the total TCO being attributed to maintenance. However, Figure 3 indicates that the cost per gram differs considerably, from 37.37 € for mesoporous alumina to 154.21 € for titanium dioxide. This variation is primarily due to differences in the quantity of material produced. This underlines the considerable differences between synthesis methods, which must be carefully evaluated when adapting a process.

**Table 2:** Cost Profiles of Cerium Oxide, Titanium Dioxide and Mesoporous Alumina

	costs (€)		
inputs	cerium oxide	titanium dioxide	mesoporous alumina
raw material	180.40	0.70	15.08
energy	0.29	16.16	14.99
labor	33.00	33.00	44.00
maintenance	0.00	1.50	1.44
accident	38.39	38.39	51.19
TCO	252.08	89.75	126.70

**Figure 3:**
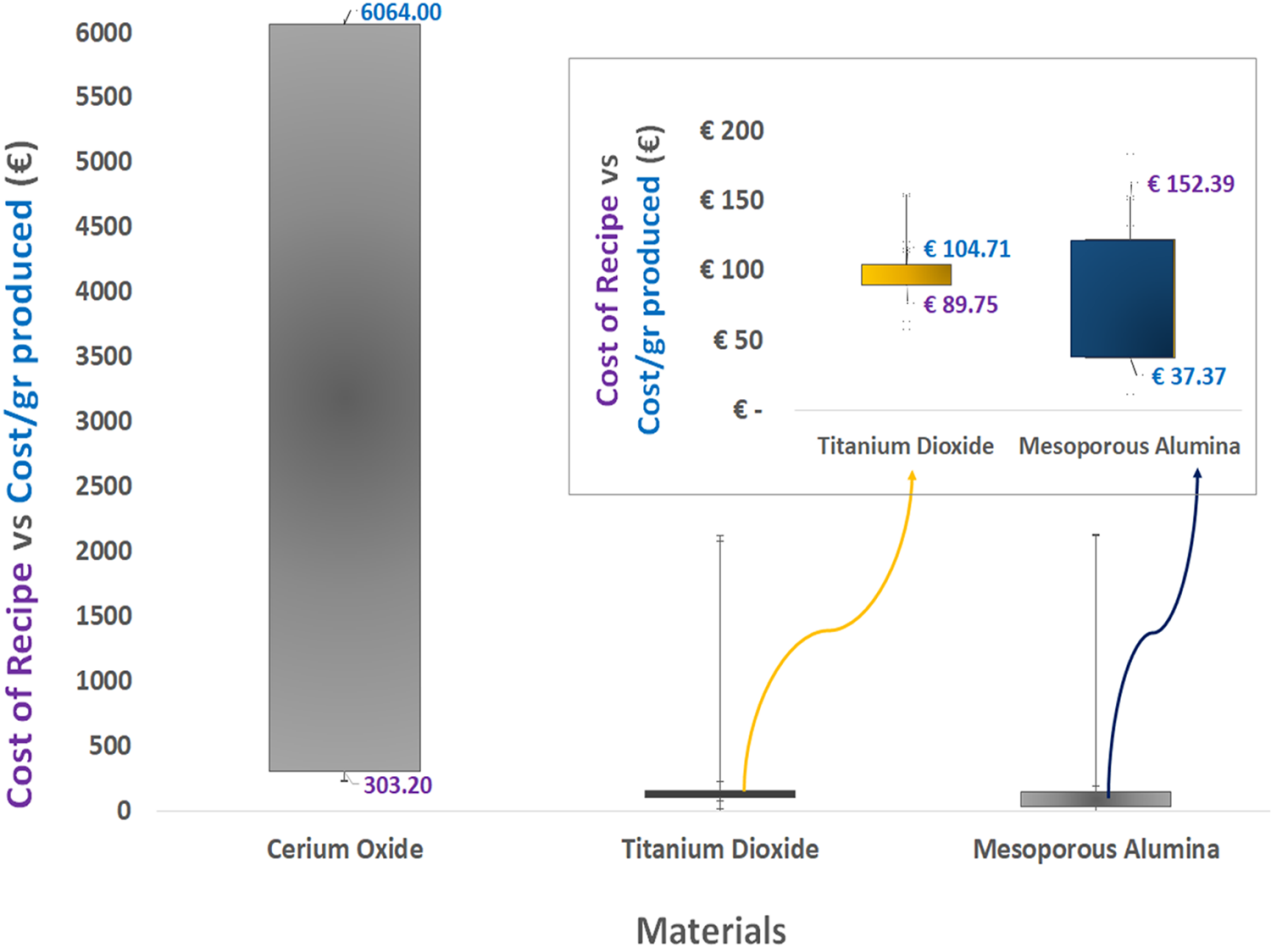
Total cost of synthesis and cost/g for cerium oxide, titanium dioxide and mesoporous alumina.

The key cost factors influencing the total cost of ownership (TCO) are similar for mesoporous alumina and titanium dioxide. However, the titanium dioxide results in the lowest overall cost, producing 4.078 g. Figure 3 summarizes the results for all the nanometals used in this study. The TCO model was applied to quantitatively evaluate the actual requirements and economic potential of various synthesis processes. Developing an accurate cost profile depends on having access to detailed and complete data.

This study developed and implemented a TCO model that moves beyond simple cost comparisons, providing a thorough and integrated view of the costs associated with different synthesis methods. Figure 4 presents the energy consumption for each process, revealing notable variations among the three materials. It is clear that the synthesis of titanium dioxide and mesoporous alumina involves more energy-intensive steps, which result in higher total energy costs. The extended processing time required for titanium dioxide, recorded at 54 h in Table 3, compared to only 1.25 h for cerium oxide, along with its lower overall yield, significantly increases energy expenses at every stage of the process.

**Figure 4:**
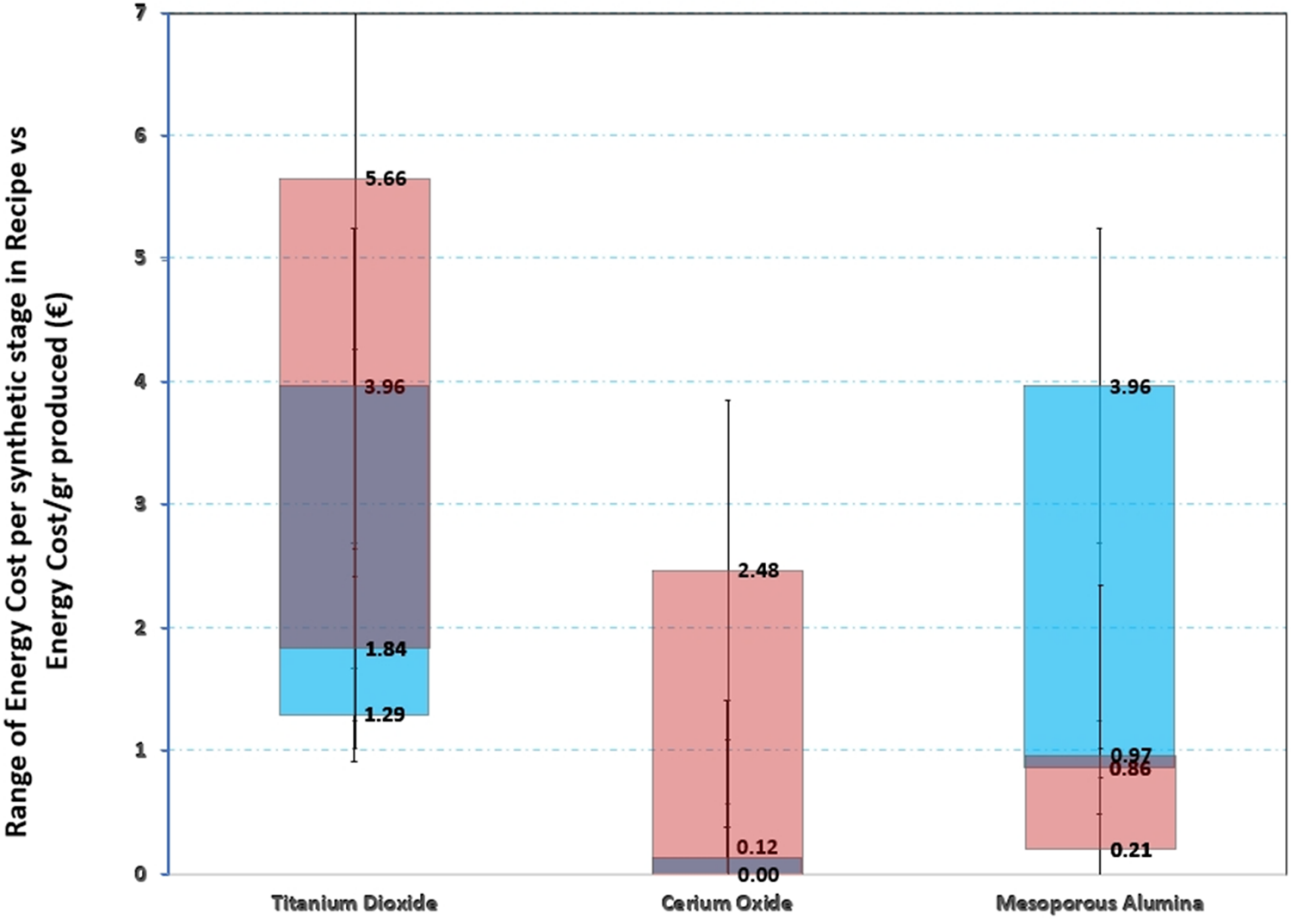
Energy cost per studied material.

**Table 3:** Overall Reaction Yield and Reaction Time Per Route

synthesis route	yield (g)	process time (h)
titanium dioxide	0.7	54
cerium oxide	0.05	1.25
mesoporous alumina	4.078	51

Although this finding may seem counterintuitive, it underscores the value of conducting early stage economic assessments to identify the most significant cost drivers. Experimentally validated synthetic routes can be compared using quantitative metrics such as yield and atom economy, as well as qualitative factors like novelty.^18^ When empirical data, such as yield, is available, it becomes possible to perform a detailed economic evaluation. Table 3 presents the process times and yields for each synthesis route. The synthesis of titanium dioxide and mesoporous alumina is disadvantaged by extended process durations, whereas the synthesis route for cerium oxide is characterized by a comparatively shorter processing time.

### Sensitivity Analysis

The three main sources of uncertainty in the cost of metal nanomaterials are directly related to variations in cost per gram. Figure 5 presents the sensitivity analysis for all three metal nanomaterials, illustrating how total cost fluctuates in comparison to the base case for each material.

**Figure 5:**
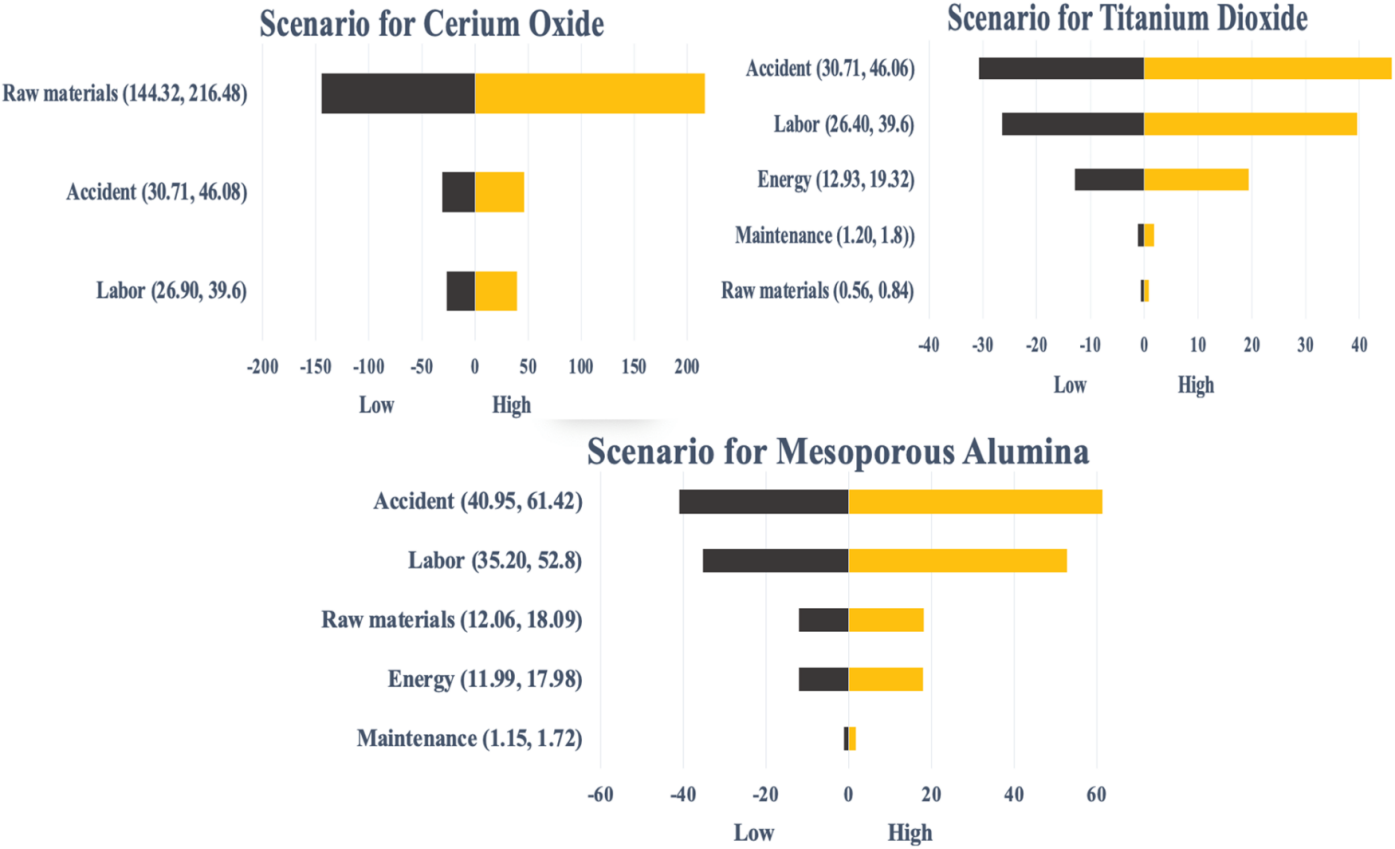
Sensitivity to different parameters on the total cost relative to base case of metal materials.

The sensitivity analysis shows that four key factors (labor, accident, and raw material costs), cause purchase price variations greater than ± 1%. Cerium oxide costs are particularly sensitive to raw material prices, with accident and raw material costs leading to changes from −3 to +14% compared to the base case. Labor and energy costs have a smaller impact, ranging from −0.02 to +2.6%. For titanium dioxide and mesoporous alumina, accident costs have the greatest influence. The analysis highlights two main strategies to improve economic viability across all nanomaterials: reducing labor and operational demands in the synthesis process, and exploring more affordable solvent options.

### Environmental Performance and Sustainability of Chemical Processes

An essential aspect in evaluating metal material synthesis is its overall “greenness.” The development of chemical reactions that are both efficient and environmentally sustainable continues to be a central goal in organic chemistry. Of equal importance is the ability to measure and compare the environmental performance of such reactions using quantitative methods.^44^ To this end, green chemistry metrics offer a framework of indicators that represent distinct facets of the principles of green chemistry. These tools allow improvements to be assessed by quantifying process efficiency and environmental burden. Because no single metric can fully describe the sustainability of a chemical process, a set of complementary indicators must be applied.^45^

### Green Metrics

The example provided laid the groundwork for the first application of green chemistry metrics to metal oxide syntheses, allowing their relative greenness to be assessed. As shown in Table 4, and Figure 6 comparative metrics for metal oxides revealed the inherent complexity of the problem. In the present work, a range of green chemistry indicators were determined, namely percentage yield, stoichiometric factor, atom economy, and reaction mass efficiency. Atom economy was used to analyze both routes by providing a straightforward method for evaluating atomic efficiency. Atom economy, alongside a reduced step count, remains fundamental to improving efficiency and minimizing waste, qualities that are straightforward to assess.^46^ A key strength of atom economy lies in its applicability without the need for experimental validation, making it an invaluable tool for rapidly predicting and evaluating waste generation in alternative routes to a target molecule.^9^ The results revealed differences in optimal conditions between the two processes, particularly in terms of atom economy and product yield.

**Table 4:** Green Metrics

material	AE (%)	percentage yield (%)	stoichiometric factor	Kernel’s RME (%)	Curzon’s RME (%)
TiO_2_	19.37	97	8.51	18.79	0.73
Al_2_O_3_	19.40	95	25.77	18.43	2.16

**Figure 6:**
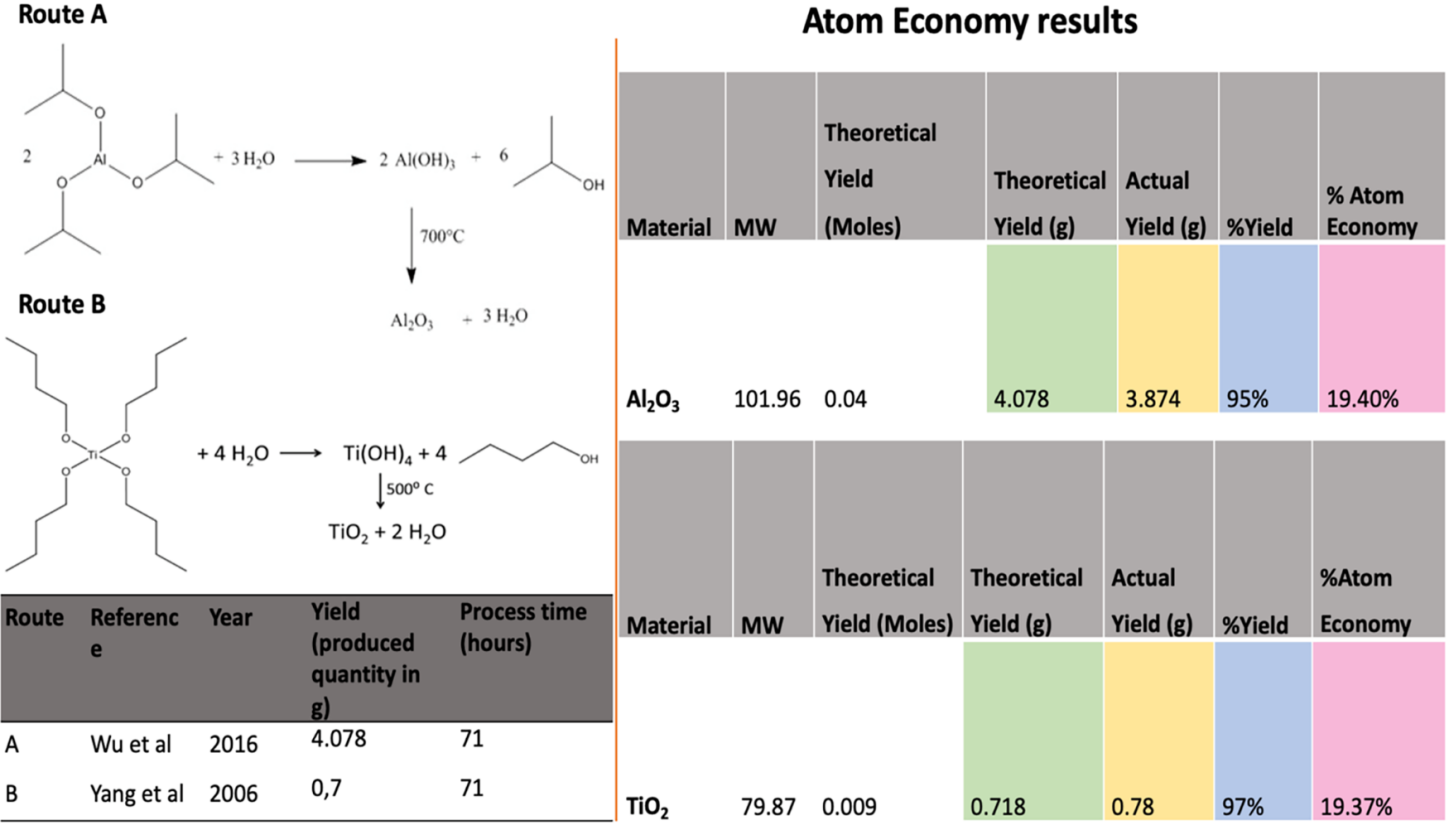
Atom economy results for two metal oxide materials.

A comparative assessment of green metrics for TiO_2_ and Al_2_O_3_ reveals that TiO_2_ demonstrates superior environmental performance in key areas relevant to green chemistry. While both materials exhibit comparable atom economies (TiO_2_: 19.37%; Al_2_O_3_: 19.40%), TiO_2_ achieves a higher percentage yield (97 vs 95%) and significantly outperforms Al_2_O_3_ in terms of stoichiometric factor (8.51 vs 25.77), indicating more efficient use of reactants and reduced chemical waste. Additionally, TiO_2_ shows a marginally higher Kernel’s Reaction Mass Efficiency (RME) (18.79 vs 18.43%), further supporting its favorable green profile. Although Al_2_O_3_ records a higher Curzon’s RME (2.16 vs 0.73%), the overall evaluation underscores TiO_2_ as the greener material, particularly when emphasizing stoichiometric efficiency and reaction yield – two metrics of central importance in green chemistry frameworks. The results illustrate the importance of directing reaction design toward greener practices, where careful control of stoichiometric balance facilitates increased reaction mass efficiency.

In recent years, numerous studies have emphasized the sustainability performance of laboratory-scale nanomaterial production. While many nanomaterials have been investigated, significant challenges remain before their widespread adoption, particularly in reducing costs^47^ and mitigating the environmental impacts of synthesis methods, both of which are central to sustainable chemistry and engineering.^48^ Sustainability assessment encompasses a comprehensive analysis of environmental, social, and economic dimensions.^49^ Yet, balancing sustainability with economic viability is a complex challenge. Material quality is not solely determined by its performance, but also by the sustainability and economic feasibility of the overall production process. In this sense, sustainability itself can be considered a marker of product quality.^50^

Earlier investigations into sustainability and economic viability considered the green synthesis of rGO nanocomposites. A central feature of this strategy involved the use of guarana as a natural reducing agent, combined with pomegranate biomass, to substitute conventional reagents with biodegradable alternatives, thereby conferring ecological and economic benefits. To quantitatively analyze the resource demands and economic potential of different synthetic approaches, we implemented a total cost of ownership-activity-based model.The most significant characteristic of this synthetic approach is its integration of green chemistry concepts with activity-based costing, which collectively promote higher yields and greater sustainability throughout the critical process steps. From an economic perspective, the streamlined method demonstrated a significant cost reduction, achieving 19.48 €/g (with three steps) compared to 248.64 €/g for the conventional approach (with eight steps). This difference is largely attributed to reduced consumption of chemicals and energy. Overall, the experimentally driven cost analysis provided valuable insights into the factors influencing total synthesis cost, helping to inform the selection of the most efficient rGO nanomaterial production route.^19^

Although there has been increasing interest in novel synthetic methods for metal nanoparticles,^51–54^ metals and metal oxides have not been sufficiently examined through the lens of Green Chemistry principles. This gap largely stems from the fact that many studies approach synthesis from the standpoint of novel phenomena, often without clearly addressing end-use considerations.^55^ This study is pioneering in scope, as it integrates green chemistry metrics with economic sustainability in the context of metal oxide synthesis. It presents a two-dimensional sustainability assessment of laboratory-scale metal oxide production processes through environmental and economic analyses. The results highlight the importance of adopting a holistic perspective when evaluating sustainability. The environmental and economic performance revealed that titanium dioxide demonstrated the strongest performance by substantially reducing the ecological footprint of metal oxide synthesis and emerged as the most feasible option.

### Benefits

A clear advantage of our methodology is its ability to provide insights into potential cost savings, while also highlighting the actual needs and economic potential of various synthesis routes. Drawing from the three case studies, and particularly when examining accident-related costs, a noteworthy contradiction emerges. This work aimed to test the hypothesis that common features in synthesis routes might reveal recognizable patterns. The case of cerium oxide demonstrated that raw material cost is the most influential factor affecting its total cost of ownership (TCO). In contrast, for mesoporous alumina and titanium dioxide, accident and labor costs were the primary cost drivers, with raw material expenses playing a minimal role. These differences point to the importance of tailoring cost-reduction strategies to the specific material and synthesis process. Our modeling results propose different ways to minimize synthesis-related costs. For example, implementing safety training programs could significantly reduce accident-related expenses. Conducting the reactions at room temperature helps lower energy consumption and minimize safety risks compared to continuous high-temperature methods. Additionally, using water as a solvent can decrease both material costs and process hazards. An important observation from this analysis is the absence of a consistent relationship between cost factors across all materials. This suggests that synthesis costs are strongly dependent on both the material type and the specific synthesis approach, a point that merits further investigation.

## Conclusions and Future Prospects

In this study, an economic model was developed and applied to various metal nanomaterials to assess their economic feasibility. Sensitivity analysis revealed that accident costs and raw material costs significantly influence the economics of synthesis. The economic evaluation indicates that understanding the primary cost drivers can make nanoparticle synthesis more cost-competitive, paving the way for sustainable synthesis and commercialization. Early stage economic analysis demonstrated that investing in safety training can remove economic barriers and enhance sustainability benefits compared to a lack of training. A sensitivity analysis was conducted to evaluate the impact of different input parameters, showing that accident costs are the largest contributor for most nanomaterials, with potential cost variations ranging from approximately −3 to +14% relative to the baseline scenario. The economic framework presented here could be extended to other nanomaterial synthesis processes. Conducting early stage economic assessments of synthesis cost factors provides valuable insights for informed decision-making during scale-up. By integrating the ABC method, TCO model, and green metrics (including percentage yield, stoichiometric factor, atom economy, and reaction mass efficiency), this study highlights the importance of combining experimental and economic data to effectively reduce overall synthesis costs.

## Supplementary Material



## Notes

Table S1. Maintenance costs; Table S2. Maintenance cost per synthesis process; Table S3. Accident cost; Table S4. Accident cost per synthesis process; Table S5. Labor hours; Table S6. Analysis of atom economy of titanium dioxide; Table S7. Calculation of limiting reactant for titanium dioxide; Table S8. Analysis of atom economy of mesoporous alumina; Table S9. Calculation of limiting reactant for mesoporous alumina; Table S10. Calculation of stoichiometric factor and Curzon’s RME for titanium dioxide; Table S11. Calculation of stoichiometric factor and Curzon’s RME for mesoporous alumina (PDF)
