# Supplementary material for: Cost Evidence Yields the Viability of Metal Oxides Synthesis Routes
Source: ACS Sustain Chem Eng. 2025 Oct 7;13(41):17370–9. doi: 10.1021/acssuschemeng.5c06752 (PMC12541911; doi:10.1021/acssuschemeng.5c06752)
Supplement: Supplementary file 1 [file sc5c06752_si_001.pdf]

Cost evidence yields the viability of metal oxides synthesis routes

Despina A Gkika[1*] and George Z. Kyzas[1*]

[1] Hephaestus Laboratory, School of Chemistry, Faculty of Sciences, Democritus University of Thrace, GR 65404 Kavala, Greece; degkika@chem.duth.gr *(D.A.G.)*, *kyzas@chem.duth.gr (G.Z.K.)*

*Corresponding author: kyzas@chem.duth.gr (G.Z.K.); degkika@chem.duth.gr (D.A.G.), *Hephaestus Laboratory, School of Chemistry, Faculty of Sciences, Democritus University of Thrace, GR 65404 Kavala, Greece, Tel.: +30-2510-46-2218*

Number of Pages: 5

Number of Figures: 0

Number of Tables: 11

Table S1. Maintenance costs.
Table S2. Maintenance cost per synthesis process.
Table S3. Accident cost.
Table S4. Accident cost per synthesis process.
Table S5. Labor hours.
Table S6. Analysis of atom economy of titanium dioxide.
Table S7. Calculation of limiting reactant for titanium dioxide.
Table S8. Analysis of atom economy of mesoporous alumina**.**
Table S9. Calculation of limiting reactant for mesoporous alumina.
Table S10. Calculation of stoichiometric factor and Curzon's RME for titanium dioxide.
Table S11. Calculation of stoichiometric factor and Curzon's RME for mesoporous alumina.

Supporting Information

Table S1. Maintenance costs.

Maintenance and Service cost	Maintenance	Service Price	Maintenance per year	Maintenance per hour
Furnace	2 years	500 €	250 €	0.029 €
Centrifuge	2 years	500 €	250 €	0.029 €
Stirring hotplate	never	-		
Ultrasonic-Cleaner	never	-		
Vacuum Oven	2 years	1000 €	500 €	0.057 €

Table S2. Maintenance cost per synthesis process.

	Yearly cost	Hourly cost	**Cerium**	**Mesoporous alumina**	**Titanium dioxide**
Furnace	250	0.028 €		0.111 €	0.166 €
Vacuum Oven	500	0.055 €		1.329 €	1.329 €
			0.000 €	**1.439 €**	**1.495 €**

Table S3. Accident cost.

Types of benefits	Subcategory	Total Value(€) (year)	Total Value(€) (1 hour)
**Recruitment (researcher) (€)**	Salary cost of replacement staff	2288	1.19 €
**Training (€)**	Retraining cost for replacement staff	2195	1.14 €
**Medical expenses (€)**	Medical & Travel expenses	1585.5	0.83 €
**Damage of property (€)**	Damage to own material/property	0	0,00 €
**Insurance benefits (€/year)**	Insurance premium	14700	7.66 €
**Human benefits (€/year)**	Salary cost of Injured employee	1500	0.78 €
**Other benefits (€/year)**	Cleaning , managerial costs	2304	1.20 €

Table S4. Accident cost per synthesis process.

	Synthesis duration (hours)	Adjusted synthesis accident cost (€)
Cerium	3	38.39
Titanium dioxide	3	38.39
Alumina	4	51.19
Cerium	3	12.36
Titanium dioxide	3	12.36
Alumina	4	16.49

Table S5. Labor hours.

**Material**	**Actual hours**	**Total hours**
Cerium oxide	3 hours	48 hours
Mesoporous alumina	4 hours	72 hours
Titanium dioxide	3 hours	72 hours

Table S6. Analysis of atom economy of titanium dioxide.

	**Titanium dioxide**			
	**Reagents**	**MW**	**Weight used (g)**	**Moles used**
**A**	Ti(OCH2CH2CH2CH3) 4	340.32	3.39g (3ml density 1.13 g/mL at 25°C of TBOT)	0.00996
	CH3CH2OH	46.07	55.25 g (70 ml)	1.19900
**B**	H2O	18.02	102.5 g (102.5 ml)	5.68800
	**Compound**	**MW**	**Theoretical yield (moles)**	**Theoretical yield (g)**
**C**	TiO2	79.87	0.00996	0.79560

Table S7. Calculation of limiting reactant for titanium dioxide.

	**Multiplier**	**Quantities**	**Option 1**	**Option 2**
A	1	0.00996	0.00996	1.42200
B	4	5.68800	0.03984	5.68800
C	1		0.00996	1.42200
		Limiting reactant is Titanium butoxide		

Table S8. Analysis of atom economy of mesoporous alumina.

	Mesoporous Alumina			
	**Reagents**	**MW**	**Weight used (g)**	**Moles used**
**A**	Al[OCH(CH3)2]3	204.24	8.3363	0.04
	HNO3 65%	63.01	0.18903	0.003
	pluronic F127	1050	17.36	0.017
**B**	H2O	18.02	72.08	4
	**Compound**	**MW**	**Theoretical yield (moles)**	**Theoretical yield (g)**
**C**	Al2O3	101.96	0.04	4.078

Table S9. Calculation of limiting reactant for mesoporous alumina.

Stoichiometry: 2*A+3*B=C				
	**Multiplier**	**Quantities**	**Option 1**	**Option 2**
A	2	0.04	0.04000	2.66667
B	3	4	0.06000	4
C	1		0.04000	2.66667
Limiting reactant is Al[OCH(CH3)2]3				

Table S10. Calculation of stoichiometric factor and Curzon's RME for titanium dioxide.

	**Stoichiometry**	**Actual mass of reactants used**	**Stoichiometric mass of reactants**	**SF**		
**Titanium dioxide**	A+4*B=C	105.89000	4.10792	25.77		
	**Stoichiometry**	**Atom economy**	**Yield**	**SF**	**Curzon's RME (%)**	Kernel's RME (%)
	A+4*B=C	0.19370	0.97000	25.77	0.73	**18.79**

Table S11. Calculation of stoichiometric factor and Curzon's RME for mesoporous alumina.

	**Stoichiometry**	**Actual mass of reactants used**	**Stoichiometric mass of reactants**	**SF**		
**Mesoporous alumina**	2*A+3*B=C	80.443	9.4442	8.51		
	**Stoichiometry**	**Atom Economy**	**Yield**	**SF**	**Curzon's RME (%)**	Kernel's RME (%)
	2*A+3*B=C	0.194	0.95	8.51	2.16	**18.43**
